# Population density drives increased parasitism via greater exposure and reduced resource availability in wild red deer

**DOI:** 10.1017/S0031182025100516

**Published:** 2025-06

**Authors:** Adam Z. Hasik, Shane Butt, Katie Maris, Sean Morris, Alison Morris, Richard S. Turner, Josephine M. Pemberton, Gregory F. Albery

**Affiliations:** 1 Institute of Evolutionary Biology, University of Edinburgh, Edinburgh, UK; 2 Department of Zoology, Trinity College Dublin, Dublin, Ireland

**Keywords:** density dependence, long-term study, NDVI, parasite, resource availability, spatial analysis

## Abstract

Exposure to environmentally transmitted parasites should increase with population density due to accumulation of infective parasites in space. However, resource competition also increases with density, lowering immunity and increasing susceptibility, offering an alternative pathway for density-dependent infection. To test the relationships between these two processes and parasitism, we examined associations between host density, resource availability, immunity, and counts of 3 common helminth parasites using a long-term study of red deer. We found evidence that immunity increased with resource availability while parasite counts declined with immunity. We also found that greater density correlated with reduced resource availability, and while density was positively associated with both strongyle and tissue worm burdens, resource availability was independently and negatively associated with the same burdens. Our results support separate roles of density-dependent exposure and susceptibility in driving infection, providing evidence that resource competition is an important driver of infection, exacerbating effects of density-dependent increases in exposure.

## Graphical abstract

**Figure:**
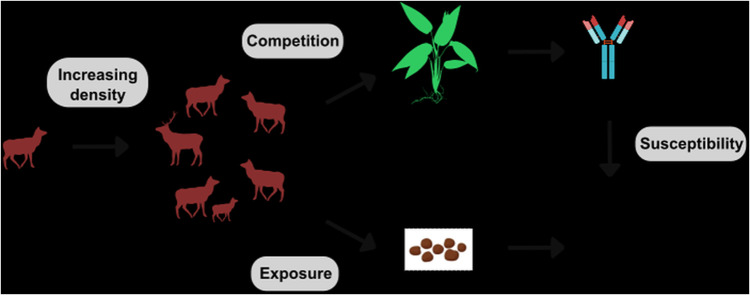

## Introduction

Parasites can impact organisms at every trophic level, affecting individual fitness and interactions with other species (Ostfeld et al., 2018; De Lisle and Bolnick, 2021; Acerini et al., 2022; Hasik and Siepielski, 2022a; Hasik et al., 2023). As such, parasites can play an important role in affecting the size of host populations (Washburn et al., 1991; Hudson et al., 1998; Morand and Deter, 2009). In addition to being widespread, parasites are unevenly distributed in space and time, such that host populations vary in both prevalence (i.e., proportion of host population parasitized) and intensity (i.e., mean number of parasites per infected host) of parasitism that they experience (Poulin et al., 2011; Gehman et al., 2017; Hasik et al., 2024). Such variation also occurs on the local scale, with fine-scale spatiotemporal variation in parasitism *within* host populations (Coltman et al., 1999; Forbes et al., 2012; Albery et al., 2022b). As a result, any parasite-mediated effects on host populations may also vary in space and time, which presents a challenge for quantifying an overall effect of parasites on host population dynamics. An important first goal to understanding potential for parasite-mediated effects on host population dynamics is therefore to identify which variables best explain spatial variation in the distribution of parasites at fine spatial scales.

**Figure 1.**
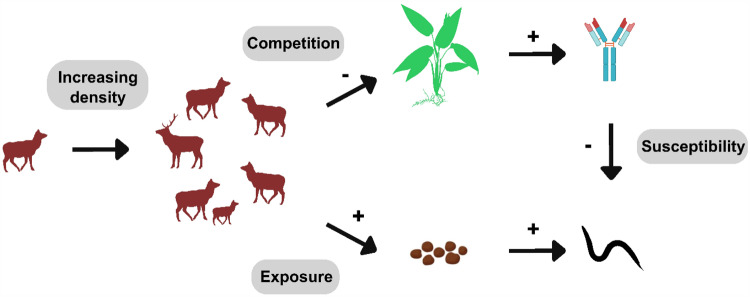
Conceptual model of the two pathways by which host density is expected to impact parasite infection. The first pathway flows from increasing host density (represented by the group of deer) to competition for resources, decreasing the amount of resources that each individual host has access to via either direct, interference competition, or indirect, exploitative competition. Since immune defences in many organisms are resource limited, such competition-mediated reductions in resource acquisition will reduce immune defences, making hosts more susceptible to parasite infection. The second pathway flows from host density to the environment. For parasites with an environmental transmission stage, increasing host density can increase the number of parasite infective stages, exposing hosts to more parasites and increasing infection levels.

Host density is a central driver of fine-scale variation in infection; where more individuals inhabit a given area, they tend to encounter one another and their shed parasites more frequently, driving greater *per capita* rates of infection (Arneberg et al., 1998; Detwiler and Minchella, 2009), a prediction central to understanding infectious disease dynamics (Côté and Poulin, 1995; Altizer et al., 2003) and illustrated schematically in Figure 1, lower path. However, it is important to note that there is evidence for increasing host densities protecting host populations by *decreasing* per capita infection and reducing the encounter rate (i.e., the encounter-dilution effect, Mooring and Hart, 1992; Fauchald et al., 2007; Buck and Lutterschmidt, 2017). Regardless of the specific pattern, parasite infection is a function of both exposure and susceptibility to infection (Combes, 2001; Stewart Merrill et al., 2021; Sweeny and Albery, 2022), and population density can covary with environmental drivers that could influence these effects in either direction. For example, badger (*Meles meles*) population spatial structure limits contact with areas of high parasite transmission, resulting in negative density-dependent infection rates (Albery et al., 2020a). While density-dependent transmission has been the focus of disease ecology studies for decades, empirical evidence is controversial and host–parasite system-specific (Brunner et al., 2017; Hopkins et al., 2020; Tompros et al., 2022; Hasik and Siepielski, 2022b), highlighting the need to (1) consider density–infection relationships in multiple host–parasite systems, and (2) consider alternative mechanisms by which density can influence infection – one of which is the distribution of resources (Albery et al., 2020a, 2024b).

Resource availability can covary with exposure and infection in important ways, determining the spatiotemporal distribution of infections. For example, when immunity is energetically costly, increased nutrition and/or resource availability strongly improves host immune defences (Boots, 2011; Budischak et al., 2018a; Hasik et al., 2023). In cases where immune function increases with resource availability, parasite prevalence is predicted to decrease (Becker and Hall, 2014), illustrated in Figure 1, upper path. Therefore, if high density correlates with high-resource availability, it could counteract density-dependent increases in exposure. Alternatively, if higher densities result in greater intraspecific competition that outweighs the available resources, this could result in higher-density populations that are both more exposed and more susceptible, which could drive synergistic greater infection levels in these areas (Figure 1, upper path). While other processes could drive density–susceptibility relationships (e.g., crowding increasing stress and reducing immune function), disentangling such environmentally mediated processes is key to understanding variation in parasitism (Krasnov and Poulin, 2010; Bolnick et al., 2020; Shearer and Ezenwa, 2020; Hasik and Siepielski, 2022b), and yet we have a very poor understanding of how resources, density, and infection covary and interact.

Infection could thus covary with density both positively and negatively in the wild depending on the balance of resources, competition, and foraging behavior. However, the field lacks information from studies of density–infection trends that simultaneously address exposure and susceptibility with sufficient statistical power, making it unclear how these effects manifest. Specifically, density effects are often examined by comparing multiple populations – or a population at different times – to detect whether higher densities are associated with greater infection (McCallum, 2001; Lloyd-Smith et al., 2005; Hopkins et al., 2020). Recent work on Soay sheep showed that these population-level measures of density provide distinct information about the factors driving infection (Albery et al., 2024b). In particular, correlated distributions of vegetation and host density in space and time are likely to drive divergent patterns of infection (Wiersma et al., 2023; Albery et al., 2024b). Nevertheless, it remains unclear precisely how spatiotemporal variation in resource availability compares with the effects of density on parasite exposure.

Here, we use data from a long-term study of an exceptionally well-characterized wild ungulate population of red deer (*Cervus elaphus*) on the Isle of Rum, Scotland to ask 2 questions. First, is greater resource availability associated with better condition and thus the ability to resist parasites? Resistance is a host strategy where hosts limit parasite infection levels, whereby tolerance is the strategy where hosts limit the harm associated with increasing parasite infection levels. Second, how do resource availability and host density explain counts of three common faecal-oral, environmentally transmitted gastrointestinal parasite taxa? Although the population is known to be heavily spatially structured both in terms of the distribution of parasites (Albery et al., 2019) and deer (Clutton-Brock et al., 1982; Albery et al., 2021b), we have yet to investigate how spatial structuring of parasitism might emerge from the distribution of population density, and how the effect of density might act via greater exposure compared to greater susceptibility via the effects of resource availability. We expected that host condition would increase with resource availability, higher individual parasite counts would be associated with greater density (through increased exposure), and, independently, with density-driven reductions in resource availability (through increased susceptibility via reduced immune defences). Untangling these two processes would help to identify a central cost of density for parasitism, expanding our understanding of density-dependence to encompass variation in resource availability.

## Material and methods

### Study system

**Figure 2.**
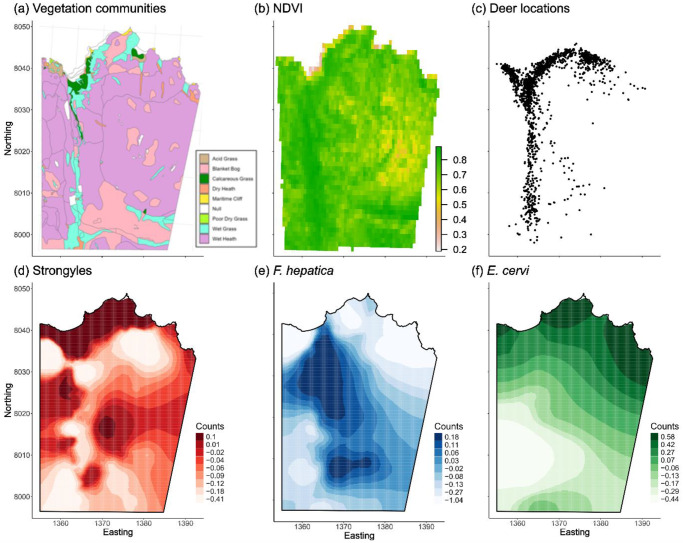
Spatial distribution of vegetation communities (a), NDVI (b), and deer locations (c) in addition to strongyle (d), *F. hepatica* (e), and *E. cervi* (f) parasite counts throughout the study area, when considering data from all deer. (a) shows the vegetation communities in the study area. Note that the high-quality vegetation communities (specifically calcareous grass in dark green and wet grass in turquoise) overlap neatly with the greater values in (b) which denote greater availability of vegetation. Points in (c) represent the centroid of the estimated annual home range for each individual deer across all years. Shown in the bottom row are projections of the spatial distribution of the raw data for parasitism across all individual deer and all years (providing a representation of where parasite counts are more abundant). Shading of the map denotes the lower bounds of nine quantiles of the spatial effects on the link scale, rounded to two decimal places, with darker colors representing higher parasite counts. Easting and Northing are in units of 100 m grid squares, with 10 units equalling 1 km. The river at the base of the valley runs along the 1363 easting.

Data for this study were collected from a host population of individually recognized red deer in the north block of the Isle of Rum, Scotland (57°N,6°20ʹW). Rum has a wet, mild climate and contains a mosaic of high-quality (i.e., high nutrition) grassland and low-quality (i.e., high in tannins, harder to consume) blanket bog and heath. The study area runs ∼4 km north to south and ∼3 km east to west with a total area of ∼12.7 km^2^. The deer within the study area are wild, unmanaged, and free from predation pressure. The population is censused 5 times a month for 8 months of the year along 1 of 2 alternating routes (Clutton-Brock et al., 1982), has a total of ∼250 individuals at any given time, and consists mostly of females and their recent offspring. Females have defined home ranges and live in loose social groups that often include matrilineal relatives (Clutton-Brock et al., 1982). The study area has heterogeneous vegetation (Figure 2a), and the deer are mainly found on the grasslands bordering both sides of the Kilmory River (which runs north–south through the study area) and the coastal grasslands (Figure 2c).

Since 2016, data on both the helminth parasite burden and faecal antibody measurements of the population have been acquired non-invasively by collecting faecal samples 3 times during the deer year (which begins with calving in May–June) in August (Summer), November (Autumn) and April (Spring). Note that Spring comes at the *end* of the deer year immediately before calving, and the deer are in poor condition following the harsh winter conditions. In brief, observers note individually recognized deer defecating from a distance and collect faecal samples without disturbing the deer. Samples are then placed into plastic bags to keep the samples as anaerobic as possible and refrigerated at 4°C to prevent hatching or development of parasite propagules, with subsequent parasitological examination being conducted within 3 weeks in the case of strongyles (Albery et al., 2018). Detailed methods can be found in Albery et al. (2018).

Here, we focus on the 3 most common parasites infecting the red deer: strongyle nematodes (hereafter ‘strongyles’, a mix of different species with indistinguishable eggs), liver fluke (*Fasciola hepatica*) and tissue worm (*Elaphostrongylus cervi*). Strongyles have a direct lifecycle in which infective stages contaminate vegetation via faecal pellets and are subsequently consumed by a new host (Taylor et al., 2016). *F. hepatica* (Taylor et al., 2016) and *E. cervi* (Mason, 1989) both have indirect lifecycles involving a gastropod intermediate host (dwarf pond snail *Galba truncatula* and a number of land snails and slugs, respectively). After infecting and emerging from their intermediate hosts, larval *F. hepatica* contaminate vegetation near water bodies and are consumed by the deer. In contrast, deer become infected with *E. cervi* by consuming the intermediate host itself. While strongyle infections develop quickly such that calves excrete eggs within 2–3 months of birth, *F. hepatica* and *E. cervi* have longer prepatent periods, resulting in low prevalences of *F. hepatica* for juveniles relative to adults (Figure S1).

There is strong seasonal variation in parasite counts (Albery et al., 2018). The highest counts are in April when the deer have just survived the winter and are in poorest condition, females are in the late stages of pregnancy, and green-up is only just under way. The lowest counts are in November when the deer (except males) are in good condition following their summer food intake.

### Measuring immunity

To quantify host immune defences, we measured two antibodies in faecal extracts, i.e., derived from the gut mucosa: total IgA and anti-*Teladorsagia circumcincta* L3 larval antigen IgA (‘anti-Tc IgA’). Total IgA represents general investment in mucosal immunity and can be considered a measure of host condition (Gauzere et al., 2021). *T. circumcincta* is a prominent member of the strongyle community and anti-Tc IgA is a component of total IgA and has been shown to act as a protective response against strongyles (Watt et al., 2016; Albery et al., 2020b). We quantified faecal antibody levels using a protocol modified from Watt et al. (2016) in three independent sessions; full details can be found in the Supplemental Materials.

### Measuring annual density

Census data for all months were collected for the years 2016–2023, where individuals’ identities and locations (to the nearest 100 m) were recorded. We calculated annual density using a previously described pipeline for this population (Albery et al., 2021b, 2022a) using all observations of each individual in each year. In short, we estimated the annual centroid of a given deer using all of its census records for the year. Using these data, we define the mean annual centroid as the mean of its Easting and Northing positions. This approach uses a kernel density estimator, taking individuals’ annual centroids and fitting a two-dimensional smooth to the distribution of the data, producing a two-dimensional spatial distribution of the population. Individuals were then assigned a local conspecific density value based on how many conspecifics also had their annual centroids within the same hectare in the same year. Note that this is an individual-level annual measure of density as opposed to the usual population-level metric. Figure 2c shows the mean position of each deer across all years of the study.

To ensure that our use of annual density was robust we conducted a repeatability analysis of deer density. We did so using a similar protocol to our annual density estimates, using the kernel density estimator for the annual density. We then used our census data to determine the coordinates for each sighting of a given deer in a given year, using these coordinates to extract density values from our annual two-dimensional smooth of the population. After collecting all of these data, we ran a mixed effects model in MCMCglmm (Hadfield et al., 2014) with deer ID, year, and deer ID:Year as mixed effects. Using the variance estimates for these random effects, we then estimated the repeatability for each term, finding that individuals experienced highly-repeatable densities (repeatability and 95% CI estimate for individual deer: 0.48 [0.39, 0.52]), with some differences between years (repeatability and 95% CI estimate for year: 0.08 [0.03, 0.25]) and within years (repeatability and 95% CI estimate for deer ID:year: 0.08 [0.07, 0.09]).

### Measuring resource availability

To investigate whether resource availability influences parasitism, we utilized Landsat satellite-derived measures of the Normalized Difference Vegetation Index (NDVI). NDVI values range from −1 to 1, with increasing positive values indicative of more biomass and/or greater productivity (Pettorelli et al., 2005). NDVI is calculated as follows:

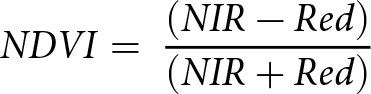


where ‘NIR’ and ‘Red’ are the respective wavelength values of the near-infrared and red spectral bands captured by the satellite’s optical sensor (Rouse et al., 1974; Pettorelli, 2013). NDVI is widely recognized as an accurate and reliable indicator of vegetation biomass and net primary productivity (Pettorelli et al., 2006, 2013), making it not only a suitable proxy of resource availability for herbivorous species, but also a useful proxy of plant biomass (Weiser et al., 1986), vegetation quality (Hamel et al., 2009), and crop yield (Fuller, 1998). Additionally, studies of arthropods (Fernández-Tizón et al., 2020), mule deer (Searle et al., 2015), and roe deer (Pettorelli et al., 2006) have shown links between NDVI and the body condition and/or biomass of herbivores, highlighting the usefulness of NDVI as a link to host susceptibility for the red deer used in this study. We calculated mean annual NDVI values (hereafter NDVI) for the deer by averaging the NDVI values from each sighting of each deer throughout a given year, giving us an estimate of the maximum amount of vegetation a given deer had access to during that year. Full details can be found in the Supplemental Materials. The spatial distribution of NDVI values aligns with the spatial distribution of high-quality vegetation in the study area (Figure 2a,b). Because host density is not likely to be independent of resource availability, we tested for correlation between these two metrics (see *Results* section).

### Does resource availability predict antibody levels and do antibody levels predict parasitism?

We used Integrated Nested Laplace Approximation (INLA) models. INLA models are a deterministic Bayesian approach, which allow for quantification of spatial effects and have been increasingly used for spatial analyses (Zuur et al., 2017; Albery et al., 2019, 2020a). We fit all models in R version 4.2.2 (R Core Team, 2021) using the R-INLA package (Rue et al., 2009; Martins et al., 2013). We focused these analyses on spring strongyle parasitism using the data from all deer, as strongyles are our best-sampled parasite taxa (see details in the next section), strongyle parasitism is most intense in the spring (Albery et al., 2018), and the dataset containing all deer is our most complete dataset. This dataset included *n* = 1,184 records from *n* = 434 deer. Results for the other seasons can be found in the Supplementary Material (Figure S2).

For the model analyzing the link between resource availability and spring total IgA we fit a gaussian model with NDVI, age category (calf, yearling, two-year-old, and adult), sex (male and female), year (categorical), and session (to control for session-specific differences in immune values) as covariates. For the model analyzing the link between spring anti-Tc IgA and spring strongyle faecal egg counts (FECs) we fit a negative binomial model with age category, sex, year, and spring anti-Tc IgA as covariates. Both of these models included individual ID as a random effect to account for repeated measures of the same individual, in addition to a spatial random effect that controlled for spatial autocorrelation.

### Do density and resources predict parasitism?

We constructed models for each of the 3 parasites in each of the 3 sampling seasons using 3 different datasets. Detailed information on sample sizes can be found in Table S1. Investigating differences among all members of the population provided information on general trends across all age categories and sexes, while analyzing juveniles separately allowed us to understand if any patterns observed in all deer manifested early in life, where parasite-mediated effects on survival are apparent (Acerini et al., 2022). Note, because of these known parasite-mediated effects on survival it is possible that we were unable to detect the greatest infection loads and/or lowest immune defense levels. Adult females make up the majority of our data and focusing on them allowed investigation of how parasites relate to reproductive success. For each of the analyses described below, we fit separate negative binomial models for FECs of each parasite in each season as we were interested in whether density and NDVI (when fit together) explain the known seasonal variation in parasitism in this system (Albery et al., 2018).

For the models analyzing all deer we first fit a base model with age category, sex, and year as covariates, while the models analyzing juveniles used the same base model structure except the age category variable only included calves, yearlings, and 2-year-olds. For the models analyzing adult females only we fit a base model with age in years (a continuous variable), female reproductive status (none, meaning she had either never given birth or did not give birth in the focal year; summer, meaning she gave birth to a calf but it died during the summer; winter, meaning she gave birth to a calf that survived at least as far as the winter), and year as covariates. In this system, female reproduction reduces immunity and increases parasitism (Albery et al., 2020b, 2021a), increased parasitism reduces juvenile survival (Acerini et al., 2022) in addition to adult survival and fecundity (Albery et al., 2021a), and as females age strongyle counts increase while *F. hepatica* and *E. cervi* counts decrease (Albery et al., 2024a), thus it is important to control for these differences in reproductive effort and age. For each of our model sets, we then added to our base models by including both annual density and NDVI.

Our focus in these analyses is on understanding if and how host density (via either competition’s indirect effects on susceptibility or density’s direct effects on exposure) explain parasitism in this population. Though each of the models described above included important covariates such as age category and sex due to their known relationships with parasitism in this system, we only present results on the relationships between annual density, NDVI, and the abundance of each of the 3 parasites for the models analyzing these datasets. The full model results can be found in the Supplemental Materials. All continuous predictors in each unique model for each dataset were Z-transformed to ensure that they were on the same scale for each analysis.

## Results

### Does resource availability predict antibody levels and do antibody levels predict parasitism?

**Figure 3.**
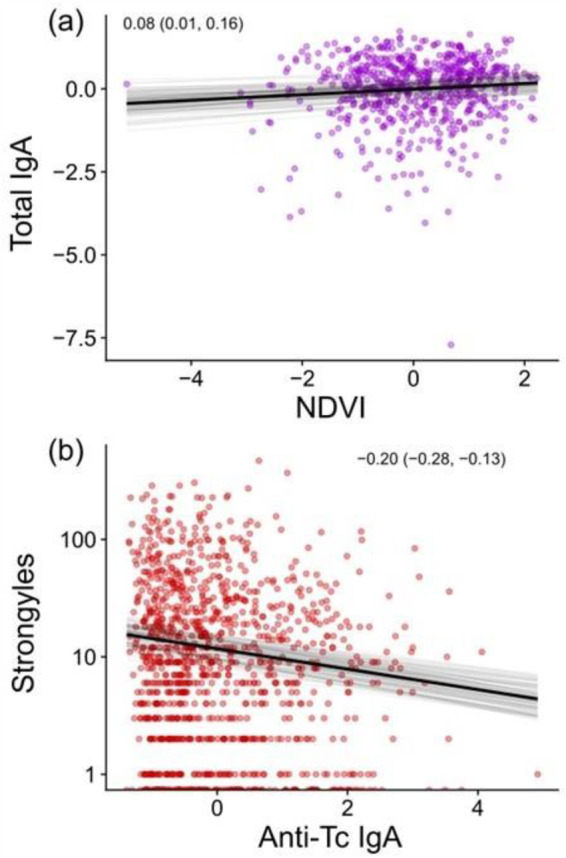
Linear regressions of the relationships between (a) NDVI and total IgA immune defences and (b) anti-TC IgA and strongyle FECs in the spring for the overall dataset containing all deer. The *x*-axes denote *z*-transformed NDVI (a) or *z*-transformed individual spring anti-TC IgA (b), with spring total IgA (a) or spring strongyle counts shown on the log scale for clarity (b) on the *y*-axis. The dark black line represents the mean of the posterior distribution for the model estimates, the light gray lines are 100 random draws from the posterior to represent uncertainty. Points denote individual samples, with transparency to allow for visualization of overplotting. The inset text in each panel represents the beta coefficients and associated 95% credible intervals from each regression.

In the overall data set, we found that variation in resources explained antibody levels, as NDVI was positively associated with total IgA in spring, the time of year when deer are in poorest condition (Figure 3a). In the same dataset we also found that spring anti-Tc IgA, a component of total IgA, was negatively associated with spring strongyle FECs (Figure 3b). These two associations were not observed in summer (Figure S2a,c), nor was there an association between NDVI and total IgA in autumn (Figure S2b) though there was a negative association between anti-Tc IgA and strongyles (Figure S2d).

### Do density and resources predict parasitism?

Mean positions of individual deer are strongly focused on the highest quality grassland, especially calcareous grassland (compare Figure 2a and Figure 2c), and these are also the regions of highest NDVI (compare Figure 2a and Figure 2b). Across the dataset for all deer, NDVI was moderately negatively associated with annual density (*R* = −0.33; Figure S3).

FECs of all three parasites varied in space across the study area (see Figure 1d–f for distributions of FECs for all three parasites combined across seasons among all deer). In general, strongyle and *E. cervi* infections were concentrated at the north end of the study area, while heavier *F. hepatica* infections tended to be more concentrated toward the middle of the study area. Supporting the findings of previous studies in this system we found that spring FECs of both strongyles and *F. hepatica* were highest in calves (Figure S4), that spring FECs of *E. cervi* moderately decreased as females aged, and that winter hinds had more parasites in the spring, highlighting the trade-off between reproduction and parasitism (Figure S5).

**Figure 4.**
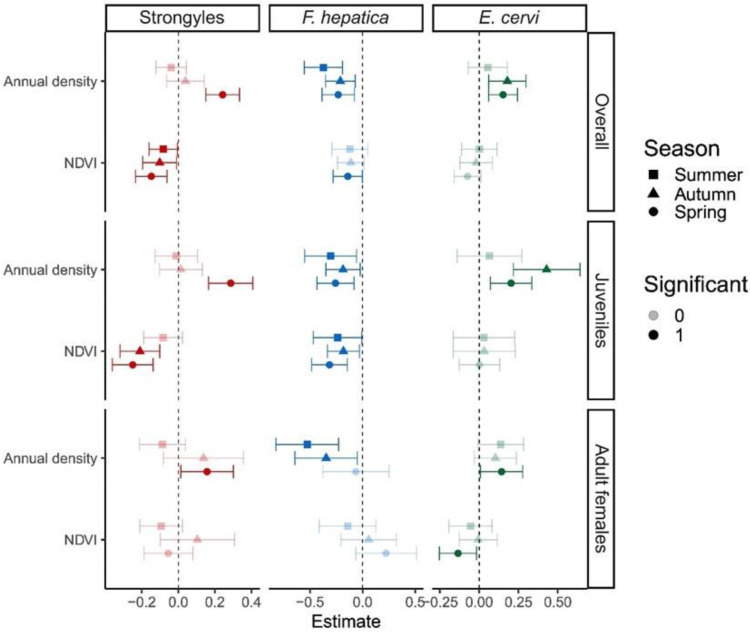
Forest plot of the relationships between individual annual density, individual annual NDVI, and parasite FECs for the datasets containing all deer, juveniles only, or adult females only, with panels for each parasite taxa and dataset. Points represent posterior estimates for mean effect sizes, error bars denote 95% credible intervals in standard deviations, color denotes the parasite taxa, and shape denotes season. Significance of the effect size is denoted by the lack of overlap of the 95% credible intervals with 0, with non-significant effect sizes faded out.

We found multiple lines of evidence supporting our hypothesis of density affecting parasitism through the twin roles of exposure and susceptibility in our analyses of strongyles in spring. Though density was not a significant predictor of strongyle FECs in the summer or autumn, it consistently predicted more parasites in the spring for all three deer datasets (Figure 4). Meanwhile, FECs of the strongyles decreased with NDVI in most seasons in the overall and juvenile datasets (the lone exception being summer FECs in the juveniles, Figure 4).

**Figure 5.**
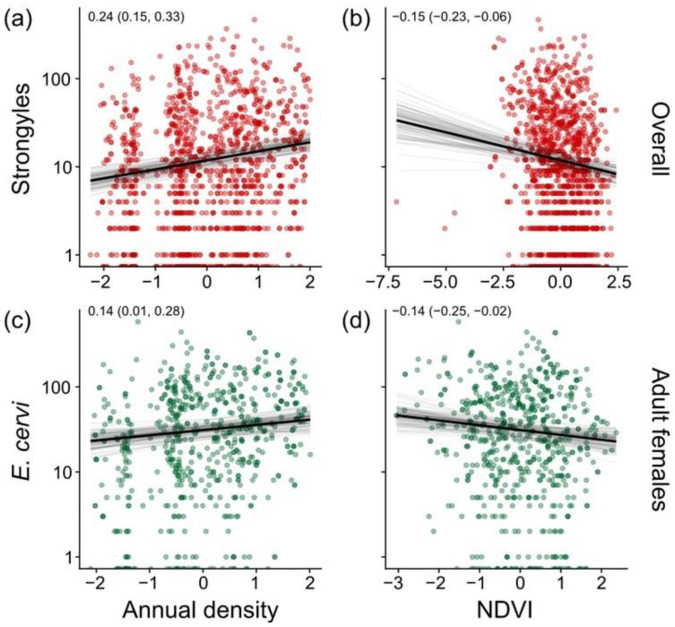
Parasite FECs regressed on annual density (left column) and NDVI (right column). the *x* axes denote *z*-transformed individual annual density (a, c) and *z*-transformed NDVI (b, d), with parasite FECs on the *y* axis shown on the log scale for clarity. Results are taken from the models regressing spring FECs on both predictors together for the overall dataset containing all deer (a, b), or the dataset containing adult females only (c, d). The dark black line represents the mean of the posterior distribution for the model estimates, the light gray lines are 100 random draws from the posterior to represent uncertainty. Points denote individual samples, with transparency to allow for visualization of overplotting. The inset text in each panel represents the beta coefficients and associated 95% credible intervals from each regression.

To better visualize these opposing effects, we plotted the linear relationships between spring strongyle FECs from the overall dataset and annual density and NDVI. Spring strongyle FECs in the overall dataset increased by 2.78 times across the range of annual density (Figure 5a), yet were 6.18 times lower at the upper range of NDVI (Figure 5b). To ensure that the negative relationship between NDVI and spring strongyle FECs in the overall dataset was not due to the influence of outliers in the NDVI data (i.e., deer seen in abnormally-low NDVI areas), we reran this model absent potential outliers. We found that the results were qualitatively the same, with spring strongyle FECs declining with NDVI (mean [95% CI] −0.15 [−0.24, −0.06]). Thus, we focus our results on the full dataset including the outliers.

We also found evidence for density affecting parasitism through exposure and susceptibility in our analyses of *E. cervi* in spring. Spring FECs increased with density in all three datasets, while the spring FECs of *E. cervi* decreased with NDVI in adult females (Figure 4). Figure 5c,d show the linear relationships between spring *E. cervi* FECs and annual density and NDVI. Beyond weak and moderate associations between autumn *E. cervi* FECs and annual density in the overall and juveniles only datasets, respectively, there were no other significant relationships between *E. cervi* and annual density or NDVI in any of the datasets (Figure 4).

In contrast to our findings for strongyles and *E. cervi*, we found moderate negative correlations between *F. hepatica* FECs and annual density, though this was dataset- and season-specific (Figure 4). Though there were no relationships between NDVI and *F. hepatica* in the adult females only dataset, we did find moderate negative correlations between NDVI and *F. hepatica* FECs in all three seasons in the juvenile dataset, as well as a weak negative correlation in the overall dataset in the spring (Figure 4).

## Discussion

Understanding whether and how parasites affect host population dynamics remains an important goal, and linking variation in the local environment and host ecology to host infection patterns provides insight into the potential for parasite-mediated effects on host population dynamics. Here, we used data from an exceptionally well-characterized ungulate system to link host density, resource availability, and immune defences to infection with three different helminth parasite taxa. We found that at the time of year when deer are in the worst condition, a measure of immune condition (total IgA) was weakly positively associated with resource availability. Replicating previous work (Albery et al., 2018) we also found that at the same time of year, strongyle FECs decreased with anti-Tc IgA (a component of total IgA). Further, we found that deer experienced reduced resource availability at higher densities, and that higher density and lower resource availability were independently associated with increased counts of strongyles in the spring for two of our datasets, with additional evidence for independent, positive associations between density and *E. cervi* counts in all three datasets, and varying, season-dependent negative associations between *F. hepatica* and density for all three datasets. These findings highlight the twin roles of exposure and susceptibility in driving infection patterns (Sweeny and Albery, 2022) and provide evidence that competition for resources and environmental confounding (i.e., hosts and parasites preferring different habitats) are likely important drivers of observed relationships between density and parasitism. This adds critical nuance to our understanding of the diverse mechanisms linking density with infection.

As hypothesized, we found strong negative relationships between NDVI (as a measure of resource availability) and parasite FECs – and in our overall dataset we found that this was because deer with access to more resources had more robust general immunity. Because immunity is costly, resource availability improves immune function in various taxa (González-Santoyo and Córdoba-Aguilar, 2012; Budischak and Cressler, 2018; Budischak et al., 2018a; Hasik et al., 2023); therefore, the resource competition associated with higher density likely undermines immune resistance in the deer, leading to higher parasite counts. These findings demonstrate that natural variation in density and its environmental effects can drive infection via susceptibility as well as via exposure, with effects that are likely multiplicative. Incorporating this variation into epidemiological models may help to accurately model the transmission and maintenance of pathogens in this and other systems. These findings are complementary to observations arising from resource supplementation in wild animal populations: generally, providing more resources drives greater contact rates, which drives increasing infection – counteracted by the improved resistance afforded by the resources and resulting immune function (Becker and Hall, 2014; Budischak and Cressler, 2018; Budischak et al., 2018a; Hasik et al., 2023). This study therefore confirms that these interactions among density, resources, immunity, and infection occur in the wild as well as being dependent on human activities.

We expected host density to increase parasitism through increasing host susceptibility as competition reduced access to resources. Supporting this hypothesis, we found that resource availability was lowest at high density (suggesting resource competition may deplete the resource availability) and host condition increased with resource availability, despite the difficulty in linking resource availability to immune defences in wild populations. Indeed, previous findings in another ungulate (Dall sheep, *Ovis dalli*) showed that nutritional condition at the individual level does not always correlate with immune defences (Downs et al., 2018). The Ideal Free Distribution (IDF) hypothesis (Fretwell and Lucas, 1970) posits that the deer should distribute themselves such that per capita resources are evenly distributed, which contrasts with our finding of low resource availability at high deer density. The IDF makes several assumptions, mainly that animals are omniscient about the availability of food in other places and that they are free to move with negligible cost, neither of which is true for the Rum deer. In the Rum population, females (and their dependent calves) live in well-defined individual home ranges and there are large stretches of unrewarding vegetation the deer would have to travel across to reach other (unknown) good patches (see Figure 2a). The deer could very well be distributed per the IDF at a local scale but we have not tested it. A study of the wild Soay sheep on St. Kilda (which has a similar environment to what can be found on Rum) provided evidence that the sheep there do not follow the IDF, even at the local scale (Jones et al., 2006). This evidence from the sheep, combined with the distribution of resources on Rum, implies that the deer do not follow the IDF.

Relationships between resource availability/acquisition and immune defences and parasitism are complex and depend on the host – parasite system considered, as increased energy acquisition by the host via the acquisition of additional resources can result in situations where either both immune defences and parasitism increase (independent energy model), immune defences increase while parasitism decreases (immune priority model), immune defences remain constant while parasitism increases (pathogen priority model), or immune defences increase while parasitism increases at first before decreasing (energy antagonism model, Cressler et al., 2014). The outcome of competition on host condition and immunity can also be complex. A study using damselfly larvae (*Enallagma signatum*) showed that although immune defences increased with resource availability, increasing competition for those same resources did not affect immune defences (Hasik et al., 2023), despite the fact that increasing competition reduced individual growth rates (Siepielski et al., 2020).

We expected that hosts in better condition would be better able to resist infection (through slowing parasite growth and limiting parasite reproduction, Nussey et al., 2014), and our analyses of spring strongyle FECs confirmed this. Namely, we found that strongyle FECs in the overall dataset strongly declined with increasing anti-Tc IgA in the spring, the time of year when infections are greatest (Albery et al., 2018). Prior work has shown that immune defences play a key role in defending from strongyle infection in this system (Albery et al., 2020b), and our results support this finding while also bolstering it by identifying a crucial connection between resource availability and overall immunity. We did not quantify fitness in this study and therefore cannot say that the deer in this population tolerate parasites (which would be evidenced by the lack of a fitness cost associated with increasing parasite loads). However, the negative relationship between anti-Tc IgA and strongyle counts, when combined with evidence for parasite-mediated fitness costs in these deer (Albery et al., 2021a; Acerini et al., 2022), suggest that resistance is involved in this case. An inability to resist infection may explain the density-dependence found for *E. cervi* in adult females if females are unable to compensate for the increased exposure to parasites that comes with the increased density in the north of the study area while also investing in their own reproduction. This observation agrees with recent findings of greater density-dependent parasitism in young Soay sheep relative to adults (Albery et al., 2024b), indicating that density-dependent infections may generally be a larger problem for weakly immune individuals across species and age classes. Otherwise, the strong positive direct effects of density on infection status in adult females (as well as the other two datasets) were likely indicative of greater levels of exposure, following the expectations of density-dependence theory (Anderson and May, 1979; Arneberg et al., 1998; Detwiler and Minchella, 2009). This effect was particularly expected for strongyles due to their direct life cycle: as deer density increases, more individuals shed larvae onto the pasture, creating higher larval abundances that then drive higher exposure rate. This represents useful observational evidence that within-population variation in density is associated with higher parasite counts. However, *F. hepatica* and *E. cervi*’s relationships may require further explanation, as both rely on an intermediate snail host to complete their life cycle.

The season-specific negative relationships between *F. hepatica* count and density (in all three datasets) and resource availability (in the overall and juvenile datasets) likely emerged from differences in habitat preference between deer and intermediate snail hosts. In the study area on Rum, the snails thought to act as intermediate hosts of *F. hepatica* are found in the stream alongside the track that runs along the western side of the study area, and deer are less dense in this area compared to the north shore because the grazing in this area is generally lower quality (Figure 2). This is the exact location where we found hotspots of *F. hepatica* infection (Figure 2), such that intermediate host distributions may explain variation in *F. hepatica* infection because of shared causality (deer seem to avoid the low-quality vegetation in the marshes, while the aquatic snails seem to prefer this habitat). This finding serves to illustrate that density-dependence of infection can be highly confounded by environmental drivers and habitat selection; this is likely to be particularly important in parasites with environmental transmission and those with intermediate hosts.

Meanwhile, *E. cervi* relies on a terrestrial intermediate snail or slug host. On Rum we believe the relevant host is the heath snail (*Helicella itala*), which only occurs in the coastal dunes in the north of the study area. This is not only where the greatest deer density occurs (Figure 2), but also where *E. cervi* counts are highest. Contrasting with *F. hepatica*, it could be that this is simply the result of both the deer and *H. itala* preferring this habitat. Another (non-exclusive) explanation is that this may be because the greater density of deer drives greater prevalence or intensity of infection in the snails, which could then increase the burden of *E. cervi* in the deer by increasing the rate at which they ingest *E. cervi* larvae. More data on the occurrence and distribution of the intermediate hosts of *F. hepatica* and *E. cervi* may better explain the spatial distribution of infection in the deer in the study area, as the incorporation of occurrence data of intermediate hosts can offer improved understanding of infection in the final host (Schols et al., 2021). This would require joint examination of spatial distributions of the host, the snails, and possibly environmental variables determining parasite development and transmission. Thus, an important next step for understanding parasitism in this system is to sample both the distribution and infection status of the aquatic and terrestrial snail hosts of *F. hepatica* and *E. cervi*, respectively.

Despite using a large amount of data from an exceptionally well-characterized system, our study has several important caveats. First and foremost, all of these data are observational, as it is impractical and undesirable for other project objectives to interfere with the natural lives of the deer. We therefore cannot necessarily identify causation in the relationships that we have detected between the various population and environmental factors and the three parasites. Another consideration is that the analyses in this study are all based on faecal propagule counts, which are an emergent property of several separate processes. Deer must first become exposed to infective larvae, those larvae must successfully establish within the host, and they must then reproduce. Internal processes such as immune defences or variation in reproductive success among the parasites, especially among strongyle species, may obscure the true infection values. However, exposure to infective stages and resource-intensive immune resistance are both expected to be important contributors to observed faecal egg counts (McKenna, 1981; Budischak et al., 2015; Watt et al., 2016), and our findings are highly supportive of the *a priori* hypotheses we set out. As such, we believe that the causal relationships we suggest to link density with infection and immunity via exposure and susceptibility are parsimonious and highly likely. Finally, though a number of studies have investigated within-host interactions between coinfecting parasites (Telfer et al., 2010; Klemme et al., 2016; Budischak et al., 2018b; Hasik et al., 2024), such an analysis was beyond the scope of this study. Linking the patterns uncovered in this study to potential within-host interactions between the many coinfecting parasites within the deer offers an exciting avenue for future study.

In conclusion, our study has revealed several important patterns relating the infection patterns of a long-lived mammal to the local biotic environment and characteristics of the host population. These results not only support prior work in other systems (Gehman et al., 2017; Bolnick et al., 2020; Hasik and Siepielski, 2022b), but highlight how important it is to compare relationships with the local environment to host metrics like population density when investigating infection. Further, by identifying the fine-scale spatial patterns of infection by 3 common helminth parasites, this study has provided an important first step toward the goal of understanding how parasites affect host population dynamics. Future studies could expand on this study by linking this variation to host fitness and survival, revealing how parasites themselves ultimately determine the distributions of host populations in space and time.

## Supporting information

Hasik et al. supplementary material Hasik et al. supplementary material

## Supplementary material

The supplementary material for this article can be found at https://doi.org/10.1017/S0031182025100516.
