# Supplementary material for: Population density drives increased parasitism via greater exposure and reduced resource availability in wild red deer
Source: Parasitology. 2025 Jul 14;152(7):724–34. doi: 10.1017/S0031182025100516 (PMC12418284; doi:10.1017/S0031182025100516)
Supplement: Hasik et al. supplementary material [file S0031182025100516sup001.docx]

**Figures S1-S5, Table S1**


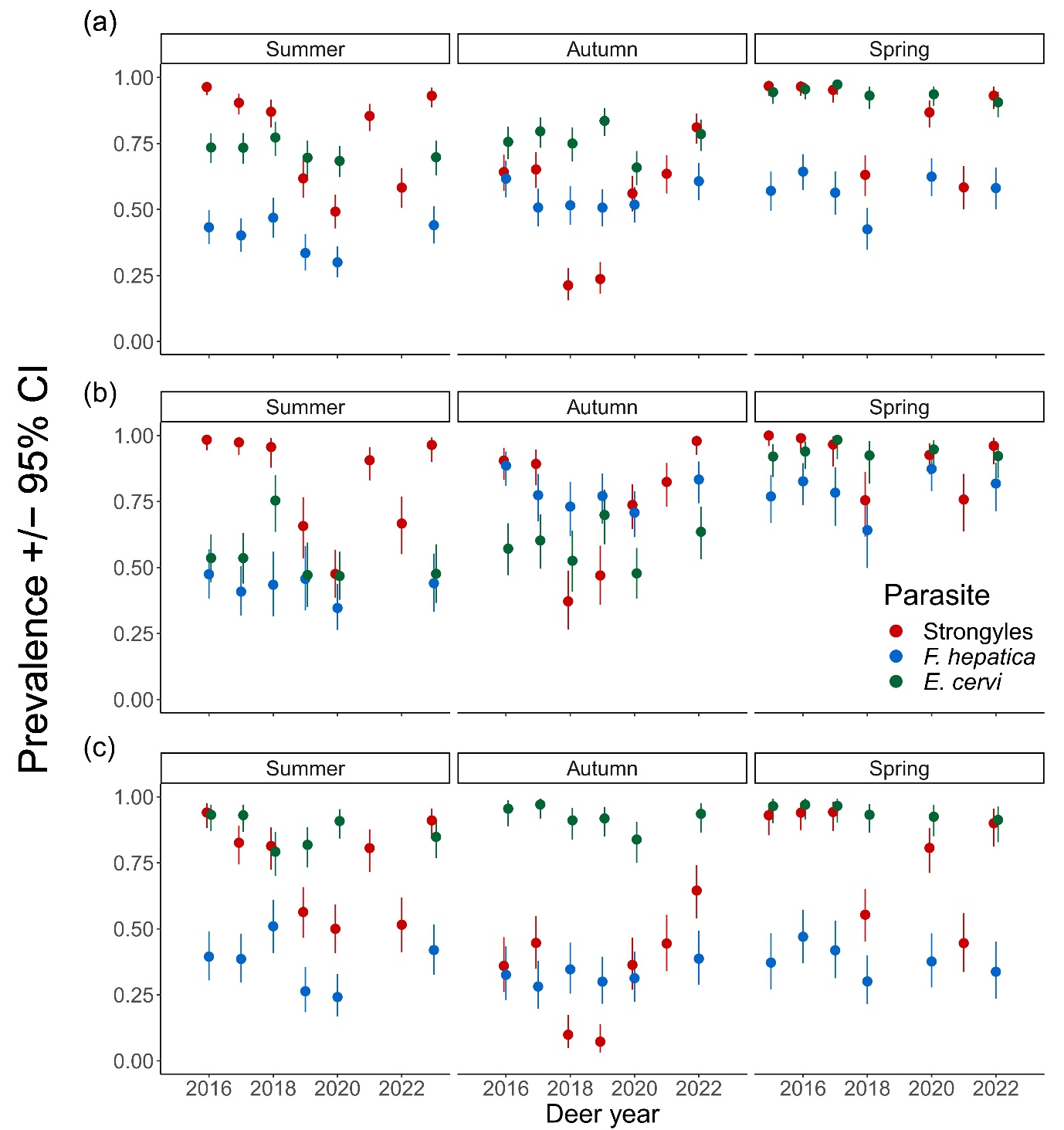
**Figure S1 -** Plots of the seasonal prevalence patterns for each parasite for (a) all deer, (b) juveniles only, and (c) adult females only, with Deer year on the x-axis. The deer year runs from May 1^st^ to April 30^th^, so deer year 2016 ran from May 1^st^ 2016, with first sampling session in summer 2016, to April 30^th^ 2017. Points represent mean prevalence values, error bars denote 95% confidence intervals, and color denotes the parasite.

**
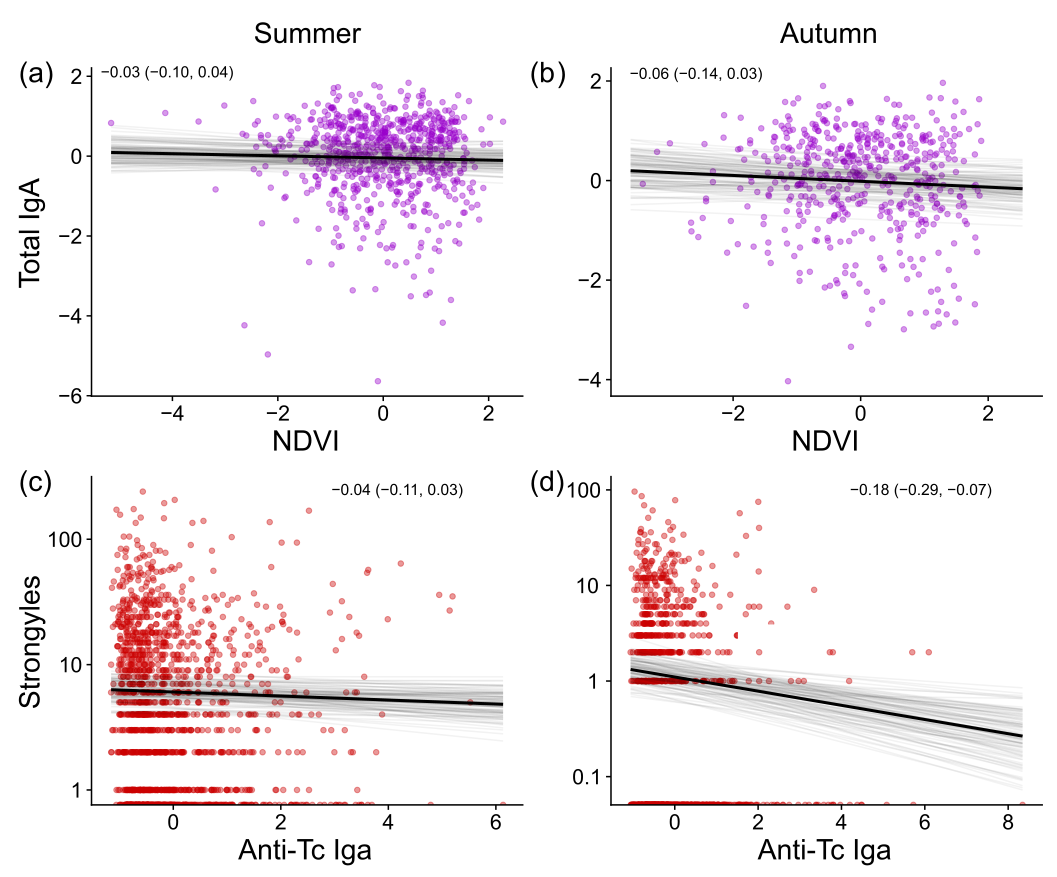
**

**Fig. S2 –** Linear regressions of the relationships between (a-b) annual max NDVI and total IgA immune defenses and (c-d) anti-Tc IgA and strongyle FECs for the overall dataset containing all deer in the summer and autumn, with columns for each season. The x axes denote mean annual max NDVI (a-b) or individual spring anti-Tc IgA (c-d), with total IgA (a-b) or strongyle counts on the log scale (c-d) on the y axis. The dark black line represents the mean of the posterior distribution for the model estimates, the light grey lines are 100 random draws from the posterior to represent uncertainty. Points denote individual samples, with transparency to allow for visualization of overplotting. The inset text in each panel represents the beta coefficients and associated 95% credible intervals from each regression.

**Figure S3 -
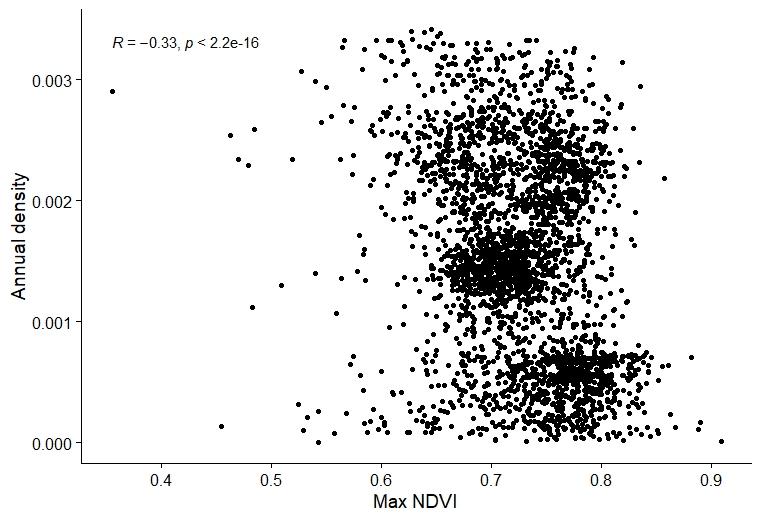
**Plot of the correlation between annual max NDVI and mean annual density, with points representing individual deer.

**
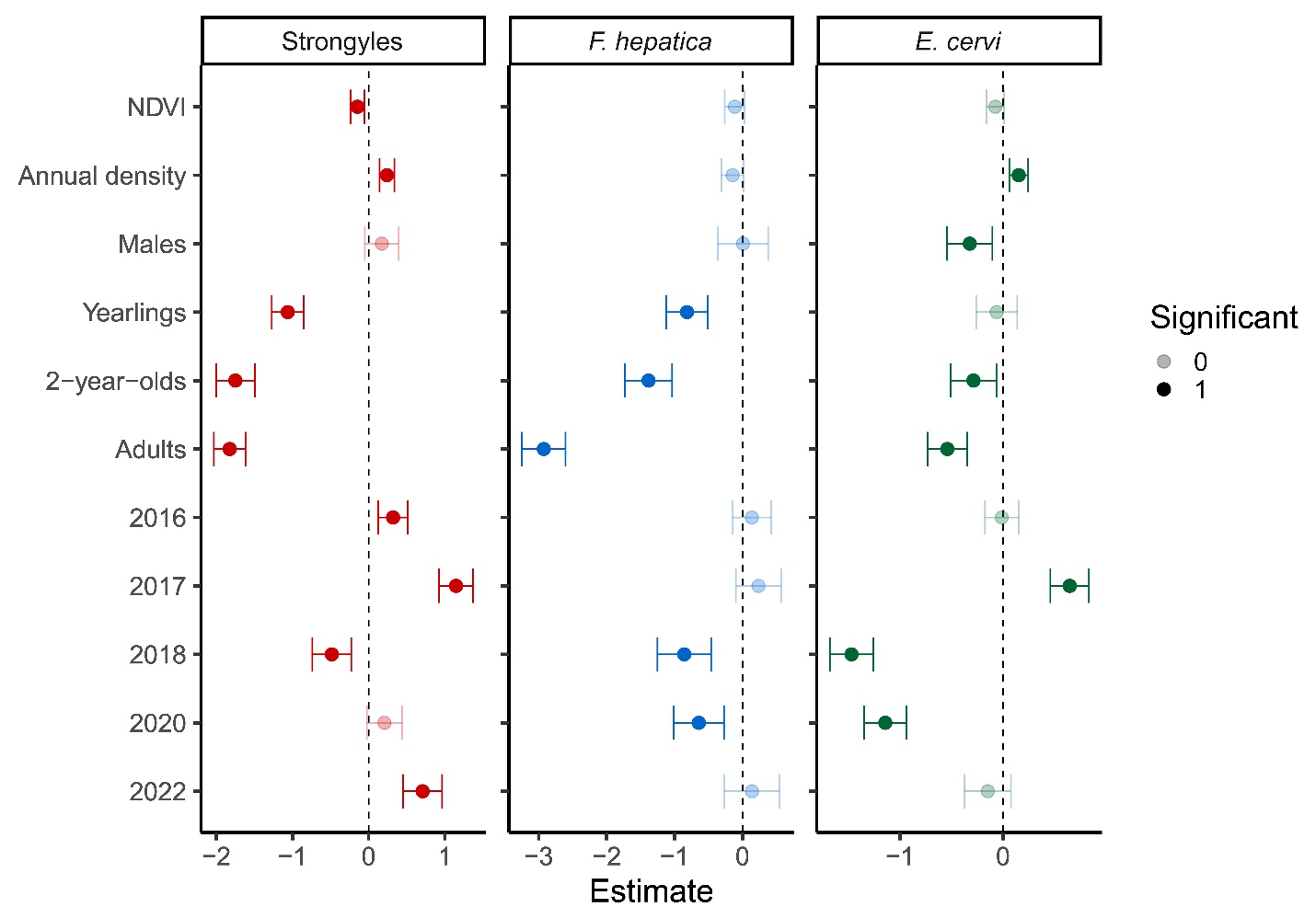
Figure S4 -** Forest plot representing the full model of the relationships between annual density, NDVI, age category, sex, and spring fecal egg counts for the dataset containing all deer, with panels for each parasite. Points represent posterior estimates for mean effect sizes, error bars denote 95% credible intervals in standard deviations, and color denotes the parasite taxa. Significance of the effect size is denoted by the shading of the points.

**
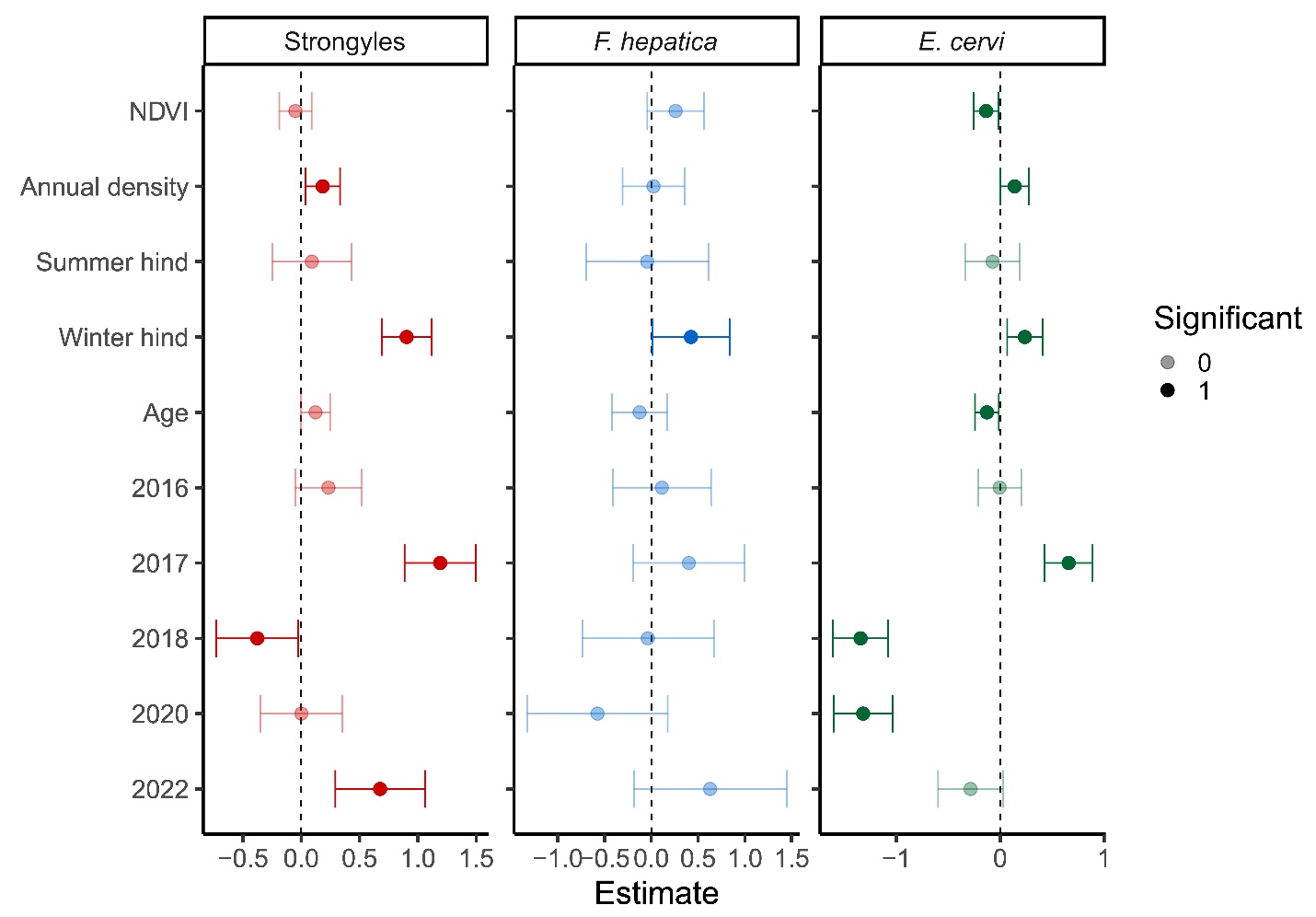
Figure S5 -** Forest plot representing the full model of the relationships between annual density, NDVI, reproductive status, age, and spring parasite counts for the dataset containing adult females only, with panels for each parasite. Points represent posterior estimates for mean effect sizes, error bars denote 95% credible intervals in standard deviations, and color denotes the parasite taxa. Significance of the effect size is denoted by the shading of the points.

**Table S1.** Information on sample sizes for the datasets broken up by age class, parasite, and season.

| Dataset | Season | Parasite | *n* individuals | *n samples* |
| --- | --- | --- | --- | --- |
| Overall | Summer | Strongyles | 674 | 1,854 |
|  |  | *F. hepatica* | 599 | 1,464 |
|  |  | *E. cervi* | 601 | 1,472 |
|  | Autumn | Strongyles | 628 | 1,720 |
|  |  | *F. hepatica* | 600 | 1,538 |
|  |  | *E. cervi* | 544 | 1,333 |
|  | Spring | Strongyles | 517 | 1,530 |
|  |  | *F. hepatica* | 503 | 1,378 |
|  |  | *E. cervi* | 463 | 1,223 |
|  |  |  |  |  |
| Juveniles | Summer | Strongyles | 515 | 825 |
|  |  | *F. hepatica* | 438 | 641 |
|  |  | *E. cervi* | 440 | 645 |
|  | Autumn | Strongyles | 476 | 798 |
|  |  | *F. hepatica* | 448 | 706 |
|  |  | *E. cervi* | 397 | 622 |
|  | Spring | Strongyles | 384 | 668 |
|  |  | *F. hepatica* | 370 | 601 |
|  |  | *E. cervi* | 335 | 541 |
|  |  |  |  |  |
| Adult females | Summer | Strongyles | 235 | 906 |
|  |  | *F. hepatica* | 224 | 705 |
|  |  | *E. cervi* | 225 | 709 |
|  | Autumn | Strongyles | 214 | 791 |
|  |  | *F. hepatica* | 208 | 704 |
|  |  | *E. cervi* | 187 | 656 |
|  | Spring | Strongyles | 220 | 787 |
|  |  | *F. hepatica* | 218 | 702 |
|  |  | *E. cervi* | 195 | 660 |

**Additional details of immune assays**

Due to logistical constraints, we quantified fecal antibodies over three sessions. Sessions 1 and 3 used samples collected from April 2016 – August 2018 and April 2021 – November 2023, respectively and we analyzed them at Ashworth labs. Session 2 used samples from November 2018 through November 2020 and were analyzed at the Moredun Research Institute.

We first extracted fecal supernatant by weighing out 0.6g (+/- 0.005g) fecal matter, which was stored at -20°C until extraction, into a clean labelled Eppendorf tube before mixing thoroughly with 0.9ml of PBS containing protease inhibitors (cOmplete™ Mini Protease Inhibitor Cocktail tablets; Roche; 1 tablet mixed with 7 ml Phosphate Buffered Saline). We left the mixture to stand for a minimum of 5 minutes, which allowed the inhibition of proteases and extraction of antibodies into the solution, and then centrifuged at 10,000g for 5 minutes. After centrifugation, we carefully pipetted the supernatant off into a second clean labelled Eppendorf tube and stored at -20°C until ready for assaying.

We measured two antibodies via fecal ELISA: Total IgA and anti-*Teladorsagia circumcincta* third larval stage (L3) IgA (anti-Tc IgA) using a method developed in sheep (Watt et al. 2016). Due to known cross-reactivity of the method for detecting anti-Tc IgA with other strongyle species (Froy et al. 2019), this assay is taken to represent a general anti-strongyle response, as opposed to a *T.circumcincta*-specific response. ELISA plates were coated with 2ug/ml sheep-derived capture antibodies for total IgA (Bethyl laboratories), and L3 antigen for anti-Tc IgA (Moredun Research Institute), at 4°C overnight. Fecal extracts were diluted at 1:64 for total IgA, and undiluted for anti-Tc IgA due to lower concentrations of antibodies. Hereafter, the ELISA protocol was carried out as described in Watt et al (2016). The dilution of fecal extracts for total IgA was determined by carrying out serial dilutions on a set of samples, and selecting the dilution at which different concentrations of antibodies had the widest spread of optical densities. For sessions two and three, we checked OD correlations for sample duplicates, and any sample duplicates where *r* < 20 were either retested, or excluded from the data. To obtain final OD values, the samples were corrected using OD values of plate control and plate blank samples according to the following session-dependent calculations:

Session 1 was Final OD = (Sample OD – mean Plate negative (blank) OD) / (mean Plate Positive (control) OD – mean Plate negative (blank) OD). All samples were run on duplicate plates. The mean value for the two duplicates, after correction, was taken for each sample and used for analysis.

Session 2 was Final OD = (mean Sample OD – mean Plate negative (blank) OD) x (mean Positive (control) OD from all session IgA or TcA plates/mean Plate Positive (control) OD). All samples were duplicated on the same plate, so mean OD value for both replicates was taken before correction.

Session 3 was Final OD = (Sample OD – mean Plate negative (blank) OD) x (mean Positive (control) OD from all session IgA or TcA plates/mean Plate Positive (control) OD). All samples were run on duplicate plates. The mean value for the two duplicates, after correction, was taken for each sample and used for analysis.

**Additional details of NDVI calculations**

We used the 'LandsatTS' package (Berner et al. 2023) to acquire Google Earth Engine-hosted Level-2 Collection-2 Tier-1 Landsat 5 (Thematic Mapper [TM]), Landsat 7 (Enhanced Thematic Mapper Plus [ETM+]), and Landsat 8 (Operational Land Imager and Thermal Infra-Red Scanner [OLI-TIRS]) satellite imagery. Throughout the study period, approximately one image of the study area was acquired per week, each with a pixel-level resolution of 30 meters. Each image was pre-processed to normalize surface reflectance and to categorize each pixel using the automated function of mask (fmask) algorithm (Zhu and Woodcock 2012). Prior to analysis, we excluded all pixels categorized as no data, cloud, cloud shadow, snow, or water, ensuring that only 'clear' ground surface pixels were used to calculate NDVI. We limited our analyses to pixels containing NDVI values of 0.15 and above, as values below this threshold typically indicate non-biomass areas such as concrete or buildings. Additionally, due to systematic differences in surface reflectance and spectral indices among Landsat sensors (Roy et al. 2016), we cross-calibrated the data among sensors to ensure that NDVI values were comparable regardless of the sensor. Cross-calibration followed the random forest model workflow of Berner et al. (2023, available from https://github.com/logan-berner/LandsatTS). In brief, the approach involved identifying the characteristic reflectance at sample sites during the growing season, defined as the beginning of March to the end of September, using Landsat 7 and Landsat 5/8 data from the same years. This was used this to train a random forest model to predict Landsat 7 reflectance from the Landsat 5/8 reflectance values. To account for a dearth of valid NDVI pixel data with which to train the model, we employed the high-latitude training dataset provided in the LandsatTS package. From these data, we again used a process from LandsatTS package by Berner et al. (2023) to quantify the growing season characteristics. This process involved iteratively fitting cubic splines to pixel measurements pooled over a seven-year moving window within the growing season. Observations were exponentially-weighted by distance in years from the focal year, so that observations from the focal year were most important in calculating its spline. Outliers were excluded and the splines refitted until all observations were within a 30% bound of the fitted spline. If there were fewer than ten observations in the focal window, the spline was not fit. We then estimated the maximum NDVI and the associated day of year for each pixel from its fitted spline. We calculated mean annual NDVI values (hereafter NDVI) for the deer by averaging the NDVI values from each sighting of each deer throughout a given year, giving us an estimate of the maximum amount of vegetation a given deer had access to during that year, giving us an estimate of the maximum amount of vegetation a given deer had access to during a given year. Because host density is not likely to be independent of resource availability, we tested for correlation between these two metrics (see *Results*).

**Berner LT, Assmann JJ, Normand S and Goetz SJ** (2023) ‘LandsatTS': an R package to facilitate retrieval, cleaning, cross-calibration, and phenological modeling of Landsat time series data. *Ecography* **2023**(9)**,** e06768. <https://doi.org/https://doi.org/10.1111/ecog.06768>.

**Froy H, Sparks AM, Watt K, Sinclair R, Bach F, Pilkington JG, Pemberton JM, McNeilly TN and Nussey DH** (2019) Senescence in immunity against helminth parasites predicts adult mortality in a wild mammal. *Science* **365**(6459)**,** 1296-1298. <https://doi.org/https://doi.org/10.1126/science.aaw5822>.

**Roy DP, Kovalskyy V, Zhang HK, Vermote EF, Yan L, Kumar SS and Egorov A** (2016) Characterization of Landsat-7 to Landsat-8 reflective wavelength and normalized difference vegetation index continuity. *Remote Sensing of Environment* **185,** 57-70. <https://doi.org/https://doi.org/10.1016/j.rse.2015.12.024>.

**Watt KA, Nussey DH, Maclellan R, Pilkington JG and McNeilly TN** (2016) Fecal antibody levels as a noninvasive method for measuring immunity to gastrointestinal nematodes in ecological studies. *Ecology and Evolution* **6**(1)**,** 56-67. <https://doi.org/https://doi.org/10.1002/ece3.1858>.

**Zhu Z and Woodcock CE** (2012) Object-based cloud and cloud shadow detection in Landsat imagery. *Remote Sensing of Environment* **118,** 83-94. <https://doi.org/https://doi.org/10.1016/j.rse.2011.10.028>.
